# Carceral Experiences of White-Collar Offenders: Qualitative Research Design Utilising the Offender-Based Definition and Pierre Bourdieu’s Capital Theory

**DOI:** 10.1007/s10611-022-10038-x

**Published:** 2022-06-15

**Authors:** Andrzej Uhl

**Affiliations:** Faculty of Law, Heidelberg University, Heidelberg, Friedrich-Ebert-Anlage 6, Germany

**Keywords:** White-collar offenders, Capital theory, Prison sociology, Prison adjustment, Special resilience, Offender-based definition

## Abstract

As the awareness and extent of white-collar crime increases, the number of prison inmates from the middle and upper classes can be expected to grow. However, existing scholarship on the imprisoned white-collar offenders has geographical and methodological limits, is of a predominantly explorative nature and often employs definitions focused on the offence rather than the perpetrator. This study attempts to advance the current state of research by utilising Bourdieu’s capital theory in the description and explanation of the prison experience of a sample of 13 politicians, businesspersons, and lawyers serving prison terms for corruption and embezzlement in Poland. Deductive analysis of semi-structured interviews reveals how participants used social, cultural, and symbolic capital to secure an advantageous position whilst in prison. Due to varied assets such as their non-criminal identity, interpersonal skills and legal knowledge, the incarcerated elites studied were able to curry favour with guards, win recognition from fellow inmates and, unlike most prisoners, maintain supportive connections with the outside world. When considered within Bourdieu’s framework, these results provide an insight into the workings of capital in carceral settings, support the special resiliency hypothesis and explain it through differences in the social situation of inmates.

## Introduction

With the growing awareness and the vast extent of white-collar crime, an increasing number of middle- and upper-class members are expected to pass through criminal justice system, which will have to allocate more resources to the prosecution of powerful criminals in order to retain its legitimacy (Reiman & Leighton 2017). After the global financial crisis, custodial sentences for executives and entrepreneurs have already became longer and more common in the US (Stadler et al. 2013), following hardening public opinion towards white-collar crime (Unnever et al. 2008), while recent crackdowns on corruption in other parts of the world have led to spectacular arrests and convictions of recognizable political figures (Menaldo et al. 2021). In Poland, where the present study was based, the last decades have seen mayors, ministers, or even senators on remand over bribery charges (Polskie Radio 2008). Even though the state’s response to the crimes of the powerful remains inadequate and selective, the era of total impunity at the top of the social ladder appears to have ended. This means that correctional systems might soon house a growing number of these unusual inmates, who have only recently received any scholarly attention.

In response to the increasing number of white-collar offenders in prison, several studies on the subject were conducted in the United States and Great Britain that applied both quantitative (Stadler et al. 2013; Crank & Payne 2015; Logan et al. 2017) and qualitative methodologies (Benson & Cullen 1988; Hunter 2012, 2015; Button et al. 2020). This emerging academic interest in convicted white-collar offenders can be linked not only to the emerging criminology of white-collar crime as an established subdiscipline with dedicated handbooks and journals (Friedrichs 2011; Alvesalo-Kuusi & Barak 2020), but also to certain developments within prison research, which has recently dedicated more attention to the distinctive experiences of subgroups of inmates as various as, for instance, the elderly, former soldiers, and LGBT+ individuals (Mann 2016; Logan and Pare, 2017; Maycock, 2021).

From this viewpoint, it is particularly interesting to consider how representatives of the ruling social classes fare within the alien world of prison, the ‘functional equivalent of a ghetto’ (Wacquant 2001), disproportionally populated by underprivileged segments of society. By observing white-collar offenders in carceral settings, criminologists can establish how far the tentacles of social privilege reach inside total institutions cut off from the wider community. This assertion undergirds the choice of theoretical framework adopted for this study, which aims to provide a convincing explanation of white-collar offenders’ resiliency, observed in prior research, to the carceral pains (Logan et al. 2017).

The extant subject literature offers substantial evidence against the special sensitivity hypothesis and identifies several recurring motives in the narrations of incarcerated white-collar offenders. However, limitations to the existing body of knowledge can also be easily identified. Firstly, almost^1^ all available findings originate from two jurisdictions - that is the US and the UK, whose criminal justice systems are more of an exception than a generalisable examples when considered within a global framework, a state of affairs which has even been criticized within Anglo-Saxon scholarship (Hunter 2019). Secondly, available literature remains explorative in its nature and most scholars ‘focused on a blunt optic around the presence or lack of a special sensitivity hypothesis’ (Button et al. 2020). Although the hypothesis now seems untenable, the reasons behind an unexpectedly good adjustment to carceral settings were only subject to tentative explanations (Stadler et al. 2013). Thirdly, and perhaps most importantly, prior research has almost exclusively adopted offense-based definitions (Hunter 2019), which are known to produce heterogenous samples when applied to offenders instead of offences (Benson & Kerley 2000). This study aims to overcome those limitations by applying a high-quality theoretical framework to classify and explain the experience of custody in a sample of Polish politicians and businesspersons carefully selected in line with Sutherland’s (1983) original concept.

## Defining White-Collar Crime, Defining White-Collar Offenders

Unlike many other forms of crime, white-collar crime is a sociological construct, not an officially recognised legal category and, as such, does not easily lend itself to measurement (Benson et al. 2016). Its operationalisation poses a substantial challenge to researchers, who have to choose between offender-based (status-based, populist) and offense-based (patrician) definitions (Shover & Cullen 2008). The former conceptualises acts ‘committed by a person of respectability and high social status in the course of his occupation’ (Sutherland 1983), whereas the most prominent of the latter portrays white-collar crime as ‘an illegal act (…) committed by nonphysical means and by concealment or guile to obtain money or property’ (Edelhertz, 1970), which effectively defines the concept down to the point it becomes synonymous with fraud in general. Patrician definitions of white-collar crime have furthermore been subject to criticism for the attempt to force the concept back into the boundaries of positive law and disentangle it from power and privilege (Shover & Hochstetler 2006: 160; Pontell 2016), as they fail to recognise that a privileged social position enables certain forms of criminality (Braithwaite 1985). This definitional trivialisation of the term has produced concepts incongruous with the popular views as to what white-collar crime is, since the general public is unwilling to label acts of offenders lacking high status or occupational opportunities white-collar crime (Galvin et al. 2021).

Although definitional debates might have an ideological dimension (Shover & Cullen 2008), additional reasons expose the limitations of using offense-based operationalisations when considering white-collar *criminals* and their punishment. Admittedly, patrician definitions might be useful to describe certain types of criminal behaviour, e.g. that which features deceptive conduct, regardless of who perpetrates it. The problem, however, begins at the point where *offense*-based definitions are applied to decide who white-collar *offenders* are. In the Yale studies (Weisburd et al. 1991), for instance, the adopted operationalisation, focusing on violators of specified laws, led the researchers to the conclusion that white-collar offences are mostly committed by the middle class rather than the socioeconomic elite. While including bank embezzlement and petty fraud in one category can perhaps be justified in terms of criminal phenomenology, they are usually perpetrated by individuals who differ in terms of social standing and criminal career (Benson & Kerley 2000; Benson et al. 2016). Most white-collar offenders thus defined (i.e. as nothing more than perpetrators of selected offences) simply end up having blue collars (Braithwaite 1985)^2^.

Since there are more offenders convicted of petty fraud than, say, corrupt politicians who reach the sentencing stage, those least representative white-collar offenders actually comprise the vast majority of any offence-based sample. The genuine upperworld offenders ‘blend in with and become less conspicuous among their more numerous middle-class cousins’ (Shover & Cullen 2008). This proportion is further distorted in prison, since higher-status defendants are especially likely to receive a suspended prison sentence under the conditions of judicial discretion (Albonetti 1999). Nevertheless, offence-based definitions certainly appear convenient to anyone who has ever tried to access a substantial sample of convicted white-collar offenders. A researcher might be tempted to ‘widen the net’ in order to include a sufficient number of observations. This, however, jeopardises the validity of research into incarcerated white-collar offenders. As most assumptions concerning such inmates, including the special sensitivity hypothesis, were developed with high-status offenders in mind, it is paramount to include measures of social status in sampling (Logan et al. 2017).

One solution to the definitional dilemma is to consider the purpose that a given operationalisation should serve (Friedrichs 2011). Interest in imprisoned white-collar criminals stems from their perceived dissimilarity to conventional offenders (Payne 2003). In essence, what makes the prison time of white-collar offenders unique is not so much their index offence as their personal characteristics and background, which is highly atypical of prison inmates. However, even qualitative studies to date often deploy offense-based definitions to research into what is expected to be the experience of the imprisoned elite. This study aims to address this gap by experimenting with an operationalisation based on the Sutherlandian concept in order to explore the prison experience of white-collar offenders as opposed to the perpetrators of ‘white-collar crimes’ who might potentially wear blue collars.

## Theoretical Framework

Pierre Bourdieu’s theory of capital was developed as a response to the earlier economic reductionism of the term, which was thenceforth meant to explain social inequality through differences other than merely the financial standing of individuals involved in social exchange (Bourdieau, 1983). In his study of various segments of French society, the author of ‘Distinction’ analysed competition over resources other than economic means, which led him to coin the new concept of ‘capital’ that would incorporate these resources alongside the original meaning of the term. Capital in general is defined rather broadly as ‘any resource effective in a given social arena that enables one to appropriate the specific profits arising out of participation and contest in it’ (Wacquant 1998). In greater detail, Bourdieu identified four major forms of capital, subdivided into three primary forms (economic, social, and cultural capital) as well as symbolic meta-capital.

Bourdieusian theory does not entail a particular definition of economic capital, which has traditionally been synonymous with the original meaning of the term (Neveu 2018). Social capital denotes the resources that are made available by networks an individual can mobilise, such as their family, circle of friends, or formal associations (Bourdieu & Wacquant 1992). Cultural capital is further broken down into embodied (knowledge, skills, familiarity with the ruling culture), objectified (objects of cultural value, such as books and paintings, which are acquired both in economic and cultural terms), and institutionalised (formal qualifications and professional titles) capital (Bourdieau, 1983, Wacquant 1998). Any economic, social, and cultural resources can furthermore be transfigured into symbolic capital if they are perceived and recognised as legitimate thus contributing to the prestige and privilege of the holder (Bourdieu 1990).

The notion of capital is inseparable from the field concept (Bourdieu & Wacquant 1992). Fields (fr. *champs*) are defined as arenas of production, circulation, appropriation, and the exchange of goods, services, knowledge, or status, and the competitive positions held by actors in their struggle to accumulate, exchange, and monopolise different kinds of power resources (Bourdieu 1984, 1990). Positions within the field can be ranked from “dominant” to “subordinate”, according to the volume and composition of capital that an individual commands (Swartz 1997). Each field requires different forms of capital (field-specific capital) and practical sense or knowledge of the rules governing social interactions in the given arena, but these rules are also constantly negotiated by the players (Bourdieu 1977, 1984). Assets, in turn, particularly cultural ones, shape their holders’ habitus and can be said to ‘own their owners’ (Neveu 2018). Therefore, the field, its actors, and their capital are all mutually linked within the framework of social interaction and the struggle for resources.

It is the theory’s focus on non-material capital that suits prisoner society where large-scale economic competition is artificially precluded by strict rules allowing inmates to own only a few personal items. Prison might be a great equaliser, especially in economic terms (Crewe 2009), but it certainly does not lack agonistic traits. Deprived of conventional indicators of status, prisoners may compete over positions in the centre of their field using, for instance, ritualistic violence and criminal connections (Shammas & Sandberg 2016). For members of the elite, in turn, circumstances of permanent material scarcity can bring out non-economic components of social privilege.

Another aspect of Bourdieu’s theory that renders it suitable for the study subject at hand is the distinction between various ‘arenas’ in which capital manifests itself in innumerable forms. There are as many types of capital as there are fields and tokens are not necessarily transferrable between fields (Bourdieu 1998). The alleged special sensitivity of the middle class to the pains of imprisonment could be attributed to the differences between their habitus and the habitus of other inmates and thus it can also be framed as a perceived lack of field-specific prison capital. An imprisoned white-collar offender abandons the familiar field of politics or business and enters an alien market whose honoured currency and rules of exchange appear mysterious and unknown; competent legal practitioners might find themselves being nothing more than ‘bungling amateurs’ in the criminal world (Willot et al., 2001). It is unclear whether the field-specific assets valid in general society, which ensured white-collar offenders such privileged lives prior to conviction, will also be recognised by the captives and their custodians. Therefore, the study of capital in prison is by implication a study of prison as a field.

## Prior Literature: Looking Beyond The Special Sensitivity Hypothesis

Empirical research into the prison experience of white-collar offenders was preceded by the identification of the so-called special sensitivity hypothesis (Benson 1988). According to federal judges surveyed by Mann et al. (1980), middle-class defendants were psychologically fragile and thus more strongly affected by prison terms than those convicted of acquisitive street crime, who are allegedly accustomed to contact with the criminal justice system. Lacking prior contact with criminal subculture, they might find themselves among a huge group of strangers with whom they can hardly identify (Payne 2003: 100). These common misconceptions ignored a series of features associated with white-collar offenders, such as age, internal locus of control, or the level of education (Hunter 2019), which had previously been found to facilitate prison adjustment, and the hypothesis did not withstand empirical scrutiny. Not only have a series of studies refuted the special sensitivity of white-collar offenders, but the converse special resiliency hypothesis has also been put forward (Logan et al. 2017).

In terms of quantitative research, the first examination of special sensitivity has been undertaken by Stadler et al. (2013), who established that imprisoned perpetrators of economic crimes fare better or just as well as other property offenders in view of five indicators of prison hardship. Rather than being an isolated minority or releasing their frustrations (Payne 2003), they were actually more likely to find friends and reported less difficulties. Complementary research on an offense-based sample in jail, where most inmates have their first experience of a total institution, failed to produce divergent evidence (Crank and Payne 2015). Typically for studies in prison adjustment, official data on disciplinary infractions and psychological counseling was employed as indicators. Moreover, imprisoned fraudsters did not differ from other non-violent property offenders in their subjective rating of the punitiveness of prison relative to other sanctions (May and Payne 2018). Recently, the extant findings have been extended to offenders with very high social status identified in a nationally (US) representative sample of the prison population (Logan et al. 2017). Educated and affluent individuals were less likely to develop feelings of hopelessness and did not vary significantly from other inmates in terms of negative affect and mental disorder.

As white-collar incarceration is by no means a common phenomenon, the subject has been more accessible to students employing qualitative methodologies. Qualitative research to date has delivered valuable insights into the lives of those few white-collar offenders who have served time for their crimes. In their recent paper, Button et al. (2020) have listed 14 previous studies based on interviews with convicted white-collar offenders, of which, however, only four addressed prison experience. Benson’s pioneering article (1988) has shown that white-collar offenders being studied distanced themselves from prison subculture, stuck to their middle-class identities and actively neutralised the criminal label through a obsequious observance of rules and associating with other former professionals. Some white-collar offenders, who had had extensive institutional experience, saw prison as one more bureaucratic structure whose dynamics can be learned in order to secure a relatively frictionless term. Incarcerated businesspersons interviewed by Dhami (2007), compensated for negative publicity and the hostile stance of judiciary with signals of support from prison staff and fellow inmates. Available interview results by Goldstraw-White (2012) have touched on questions of the effect of punishment on deterrence and life after release. In her analysis, the ensuing adversity was broken down into financial losses, abandonment by friends, and professional banishment. Nevertheless, respect from fellow prisoners and the reinforcement of family ties were mentioned as silver linings. The recent British study by Button et al. (2020) can be considered the most extensive. The difficult experiences of the offense-based sample of 13 white-collar offenders included alienation, the hostile approach of the guards, and, in one case, a physical assault. On the other hand, participants appreciated the less strict regime of open facilities, the opportunity to help other prisoners and time off stressful work. Some positive aspects, such as sense-giving, were also highlighted by Hunter (2012, 2015), who scrutinised the content of memoirs published by prominent former prisoners. Often, incarceration was portrayed as less burdensome than the protracted and humiliating trial.

## Methods

Answering the research question adequately demanded the use of qualitative accounts which ‘focus on several different aspects of the prison experience for white-collar offenders, putting us in a good position to understand how they actually experience the impact of imprisonment’ (Hunter 2020). The target population consisted of individuals of high occupational or political status (at the time of the offence) who committed non-violent property crimes during the course of legal activities and were consequently held in custody for a period of at least one month, including pre-trial detention. To minimise the impact of carceral settings on the collection of data, the sample was limited to released participants, who were then contacted directly or through their lawyers, former colleagues, and snowball. During the recruitment stage, the powerful subjects of the study were encouraged to participate in the production of original knowledge directly relevant to their lived experience (Petintseva et al. 2020). The consistent application of the offender-based definition was prioritised over the number of observations. The final sample consisted of 13 male offenders representing a variety of professions considered elite (*Table* *1*). The apparent overrepresentation of corruption charges in fact corresponds with the image of ‘crime at the top’ in Poland and prosecutorial priorities. All the participants used to be recognisable figures in their local communities, and several of their convictions were *causes célèbres*.

**Table 1:** Composition of the sample

Name	Profession before conviction	Main charge	Time
Ignacy	City mayor	Bribe-taking	15+0
Włodzimierz	City mayor	Bribe-taking	6+25
Szymon	City mayor	Bribe-taking	5+11
Aleksander	Bank branch director	Embezzlement	30+78
Łukasz	Private entrepreneur	Embezzlement	9+0
Tomasz	Deputy mayor	Bribe-taking	4+0
Franciszek	Private entrepreneur	Embezzlement	4+32
Michał	Public prosecutor	Bribe-taking	2+12
Kazimierz	Barrister	Bribe-giving	6+7
Jan	Architect, entrepreneur	Bribe-giving	2+0
Bartosz	University professor	Bribe-taking	1+0
Przemysław	Senator, entrepreneur	Bribe-giving	15+0
Filip	City mayor	Bribe-taking	5+15

Custody time in months as a sum of pre-trial detention and the time served after conviction.

Own representation

The interview protocol consisted of around 15 questions, the most important of which were “What enabled you to function in prison?”, “How did your experience of prison differ to that of other inmates?”, and “Did you receive any support from outside prison?”. The data was collected between October 2020 and January 2021. The Covid-19 pandemic left its mark on the interviews, which had to partially be conducted via video calls. The duration of the recorded parts of the interviews varied from 43 to 158 minutes, with the median length of 83.5 minutes).

The coding of the obtained transcripts was facilitated by specialised software. The code system primarily arose from the adopted theoretical framework, which accounted for the deductive procedure used. Alongside theoretical classification, accounts were coded according to thematic categories: prison time, fellow inmates, prison guards, and family and friends outside.

## Prison time

The tenor of the narratives ranged from traumatic (Ignacy) through mixed (predominant amongst the sample) to relatively positive (Kazimierz and Przemysław) or even humorous (Tomasz). None of the subjects agreed with the statement that the process itself is punishment but the regime of pre-trial detention was described as far harsher than the actual prison term. Alongside the possible habituation effects, the difference lay in the stricter isolation of the defendants and their uncertainty about its precise duration.

The time in the actual prison wasn’t so painful considering my earlier experience of remand. In a correctional facility, I’m doing my time but at least I know when I’m leaving it. On remand, however, I didn’t have the slightest idea when I’d be out. (Szymon)

The opposition between both types of detention was all the more distinct since most of the sample served their actual sentence in open facilities, which imposed considerably fewer constraints on the daily life of their inmates and allowed for more frequent visitations and more interesting prison jobs. The subjects were particularly well equipped to make good use of these opportunities, in that they were actually visited by families and eagerly accepted the offered positions.

Even without the involvement of other inmates and prison guards, detention was accompanied by various impositions. As in prior research (Benson & Cullen 1988, Button et al. 2020), the white-collar offenders studied linked their emotional distress primarily to the first fortnight of confinement, directly after they were jolted out of their professional and familial life and thrust into radically altered social settings. Three participants confessed suicidal ideation during that period, and a further three reported some symptoms of mental breakdown.

The first two to three weeks were a real tragedy, many sleepless nights. It was unbelievable and I felt fully dissociated. (Michał)

Having overcome the initial shock of entry, the subjects found themselves confronted with the mundane impositions of detention. Nine out of thirteen were befallen by ailments of varying severity. Six emphasised poor hygiene of the facilities and three complained about the prison food - the daily food allowance amounted to €1.20. A further three pointed out the lack of privacy, including Stanisław, who had previously been in the habit of sleeping during the day and working at night but now had to switch to a 6 a.m. wake-up. The material conditions of the prison also stood in sharp contrast with their previous opulent lifestyles. Jan, a trained architect, expressed amazement at the fact that facilities of such a low standard were still in use. Kazimierz exchanged his boardroom for a cell whose walls were infested by mould each winter, while Włodzimierz was entitled to one shower a week in the scorching summer months.

I'm not saying the food was awful… but in comparison with the previous standard of living that I was torn from ... zero sanitation, and on top of that, that diet ... I'm also vegetarian, and this alone was kind of difficult. (Jan)

To make matters worse, according to Jan, ‘time seemed to be the biggest problem for the inmates’. This issue, however, was successfully tackled by all the white-collar offenders and the word ‘boredom’ was not mentioned once in the total of 16 hours of recordings. In accordance with the Polish adage ‘intelligent people do not get bored’, they quickly found constructive ways of passing the time.

Inactivity kills. One would just lie down and think. Therefore, work is also a sort of escape. I used to write a lot. I could write out two pen refills in a week. (Włodzimierz)

Many participants made frequent use of prison libraries and caught up with reading they had not managed to fit into their busy lives as professionals. Others followed the news or dedicated themselves to instrumental music. For some, their cases formed their orientation point; they studied penal procedure and composed multi-page appeals against detention. Some wrote memoirs, letters, and noted down personal reflections. Beyond their hobbies, most of the sample found a prison job. Owing to their cultural competencies and good relations with staff, they were often assigned to non-strenuous but interesting positions at the prison radio and library.

## Other Inmates

Prisoner society was a great unknown for the white-collar offenders, whose vague expectations were often based on cliches and prison movies. In anticipation of custody, Włodzimierz read memoirs of political prisoners of the 80s, while Łukasz hoped his experience of military service would assist him inside. Upon entry, they devised strategies that, on one side, employed their personal resources and, on the other side, took account of the conditions of a given facility. Successful adjustment first required a careful observation of the community of the imprisoned, especially since the white-collar offenders were unfamiliar with criminal subculture and had no earlier experience of carceral settings. To this end, they learned argot and sought to understand the existing power relations.

The first days in any such institution are spent observing 'who is who'. Then there is an attempt to adapt to it, so that you are tolerated. Because this is the basic strategy to survive without difficulties interacting with such people. (Włodzimierz)

At the same time, most of them tried to reveal as little as possible about themselves whilst disclosing enough information to avoid arousing the suspicion of the cellmates. Michał, then still a prosecutor on paper, invented a story about a ‘swindle’ and passed as a former businessperson. Others refrained from advertising their former positions, instead allowing them to be appreciated tacitly, particularly as prison society was highly intolerant of any self-aggrandisement. Some described a fine line that could not be crossed without exposing oneself to jealousy and mockery.

I was afraid of this envy. And that's why I did my best to - God forbid - never show that I was any different. And yet, of course, they saw it. But I would have been a stupid asshole if I tried to show off. (Tomasz)

Aleksander, whose musical talent was highly appreciated in prison, declined an offer to play outside the facility as he believed it would have engendered envy and alienated him from prisoner society. An atmosphere of class ressentiment resonated in a truly Bourdieusian excerpt from Łukasz’s account. Having joined the prison radio due to on his previous experience of giving interviews as an executive, he discovered that his collaboration with two colleagues would hardly go smoothly.

I sensed this rivalry. They would have loved to stab me in the back so they wouldn't have to compete anymore. I was simply better than them. Well, I spoke proper Polish, with a normal accent, without lisping in Silesian or stuttering when I read aloud. They realised this difference and that created this rivalry. They wanted to prove that they were better or, even better, that I was worse. Sounds quite childish but in prison it's not funny at all. (Łukasz)

In order to legitimise themselves in their new settings, the white-collar offenders attempted to treat the other prisoners as equals and present themselves as sympathetic and supportive fellow sufferers. At the same time, they discovered that even the most hardened criminals could not be reduced to the criminal label alone. Some of the participants were eager and understanding conversationalists and, just as in the study by Button et al. (2020), they eventually became friendly with people they would never have become acquainted with in their lives outside.

People always enjoyed talking to me as I treat every person equally. There were many great conversations inside the facility and other inmates often came up to me. (Szymon)

Once the other inmates felt they were approached without prejudice, they were encouraged to learn more about their unusual companions. The white-collar offenders were seen as a curiosity and asked to share stories from their lives in business and politics. Bartosz asserted half-seriously that he held lectures on geography before a cell audience. They were not only able to add variety to the monotonous prison life but also to offer substantial help. Formal writing skills came to be particularly appreciated by prisoners, many of whom had other ongoing criminal or family cases. Helping other inmates to compose an array of requests and complaints was perhaps the most common theme across the gathered accounts and all bar one offered the service without asking anything in return. Włodzimierz answered the question of what he gained through this, saying:

Respect, I think. That's how it worked. If you're asking about material goods, I didn't need any of those. Cause what can you get there? Fags?

Conversely, sharing material goods with inmates was not likely to win friends, and could be even shunned or lead to exploitation. The food packages sent by families could only contain basic products from the prison shop, which apparently did not suffice to distinguish their owner from other inmates.

In terms of prison violence, things rarely escalated to physical altercations although some personal conflicts required an assertive approach that would avoid either escalation or submission. ‘I’m a politician and know how to deal with people’, boasted Tomasz. While Alexander claimed to have peacefully discharged tensions that would have led a less shrewd inmate to a violent confrontation, Włodzimierz adopted some of the toughness he believed was expected of him and learned how to talk the bullies down in a way that surprised and impressed his fellow inmates.

On the other hand, you should never let yourself be mistreated, sometimes even call them names yourself. Like a dog in a pack, if you don't bark back, you get in trouble. (Franciszek)

A great deal of respect was attributed to the seriousness of charges that often reached six- or seven-digit sums. In the highly publicised cases, spectacular arrests, use of special units, and perp walks - all directed against the defendant’s public image - won them a reputation of a criminal elite within prisoner society. While some prison inmates were suspected to have invented their criminal careers, the participants’ cases were often known from media coverage.

The first thing that is 'checked out' by fellow prisoners in a correctional facility is the actual extent of criminal activity by the inmate in question. It was hardly a problem for me; my case was widely known, the newspapers reported it, they even used my full name. (Kazimierz)

Other rules applied to the islands of middle class inmates found in open facilities. Recognising each other’s innocence was the entry ticket to the groups that offered friendly advice and a refuge from the monotony of prison life. The disparate society of prison gangs, in turn, rarely came in contact with white-collar offenders, who not only remained unimpressed by their customs but sometimes saw through the clumsy attempts to resume the intricate counter-culture of communist prisons described by Kaminski (2004).

The counter-culture actually exists, that is: everybody wanted it to exist - so they tried to use this argot, to imitate these customs, but I think most of them really had no clue about it. I didn't tell them, but it seemed ridiculous to me. Anyway, let them try, if it's fun for you and distracts you from your problems. I'd rather you play this kind of game than constantly think that you don't have a wife anymore, that your kids don't want to visit you. (Tomasz)

All in all, although dealing with fellow inmates was first seen as a challenge, most white-collar offenders successfully adjusted to prisoner society, were sympathised with or even looked up to. With the exception of Ignacy, whose manifested identification with law and authority separated him from other captives, the participants did not experience physical violence and alienation as they had feared upon entry.

## Staff

With reference to the second subsystem of the institution, white-collar offenders often differentiated between educated rehabilitation officers and prison psychologists on the one side and ordinary guards on the other. The former were seen as natural allies with similar cultural codes, whereas the latter were either depicted with indifference or even compared to the street offenders in terms of intellectual capacities and manners.

The station guards preferred the company of criminals, to whom you say 'Today we're hopping on one leg,' and they would hop. If you question anything, they can give you trouble because they think you make a fool out of them and so on. (Franciszek)

Compliance with prison rules was the key to good relations with guards and hardly anyone resisted the power of the institution. Having little to win and much to lose in prison, the white-collar offenders sycophantically followed the established rules. Filip and Aleksander pointed to their past organisational experiences which allowed them to read and comprehend the rules and avoid potential infractions.

Previously, I drafted the house rules of our branch office. So once I entered another structure, it wasn‘t a problem to read the rules, understand them and obey them. The first thing I did was to study the prison rules instead of listening to what some alleged friends tell you, which can be completely wrong as well. (Aleksander)

In the case of disagreement, they attempted to make their case politely and support it with reference to specific norms, which appeared more convincing given their own allegiance to the rules. Beyond mere compliance, many participants proved useful for their facilities; Kazimierz, although banned from practicing law, assisted the administration with all sorts of legal matters while Włodzimierz helped shape the cultural program. Two others assumed semi-formal positions as mentors for juvenile delinquents, which were compensated with additional visitation hours.

When I was leaving prison, the person who was in charge of so-called 'work with prisoners' was like, 'Oh you would have the whole radio here at your disposal, you could run programs'. He had hoped I would become his right-hand man and would entertain the convicts. (Bartosz)

Gradually, they ingratiated themselves with guards and secured a the reputation of what they often referred to as a ‘normal person’, who was, in their views, someone who might be imprisoned by mistake, avoids trouble, and stands out from the ‘problematic’ population.

Soon they came to like me and noticed I‘m not a criminal but a normal man, who is trying to adjust, functions well and helps others. (Franciszek)

As such, they were able to win the trust of staff and narrow the distance between them and the officers, who stepped out of character, spoke freely with white-collar offenders and granted them minor favours. These favours included, for instance, allocation to the desired cell and also less thorough checks. One of the interviewees used this as an opportunity to smuggle documents and letters he was not supposed to keep in his cell during the investigation. Enjoying the trust of the guards anyhow, he was able to hide the files in a huge staple of papers he carried to every consultation with his lawyer, which seemed justified by the complexity of his charges. This telling example resonates with the nature of the crimes of which white-collar offenders were accused: abusing trust and exploiting organisational intricacies to get away with it.

Alongside their active engagement, the white-collar offenders studied were perceived by the staff through the lens of their social standing. Szymon suspected that some of his custodians might have been afraid that imprisoned politicians could seek revenge if back in power. Michał felt that his past role as a public prosecutor won him the sympathy of the guards who, ‘after all, work in a similar branch’.

I was clearly under an umbrella of protection, I had my cell phone available around the clock. I felt I was treated differently in every respect. Kazimierz)

About a half of the sample reported some kind of privileged treatment, such as salutations including their former title, e.g. ‘Mr Professor’, ‘Mr Mayor’. Furthermore, the white-collar offenders who curried favour with the staff could count on interesting prison jobs, such as those in the prison radio and library, and rewards, including furlough. Last but not least, good relations with the prison service bode well for the positive assessment required for conditional release, which was eventually granted to all participants who were serving a sentence.

## Family, Friends and Colleagues

While the white-collar offenders were building a certain position within their facilities, most of their social resources were based on the outside. Although prison regulations imposed strict restrictions on contact with family, which was further curtailed during the Covid-19 pandemic, most of the sample managed to preserve their marriages and the relations with relatives. With the exception of Ignacy, whose wife filed for divorce, the participants saw a clear difference between their strong familial bonds and the unstable marital situation of their cellmates who, according to Tomasz, were all abandoned by their spouses and hardly visited by their children.

All prison inmates experience one great uncertainty. The worst thing that can happen to a prisoner is learning about the infidelity of a partner, even if only hypothetical. That is, if a captive learns that his partner, perhaps (!) has begun to step out on him, then he is ready to hang himself as he cannot bear the fact that he was unable to prevent it. In any case, good relations with family were the most precious thing and helped me endure that time. (Kazimierz)

While loved ones gave the participants a clear motivation to endure and leave prison and therefore had a primarily emotional impact, the families also did their utmost to improve the material conditions of the inmates. It was often their wives who sent much-needed food packages, hired the best lawyers, and started petitions against the arrests. Strong family relations also constituted a huge advantage in the assessment process leading to conditional release.

My wife mobilised [lawyer's first name], whom she knew through a friend of hers, a judge. He went to the hearing, and that is how I was released - on bail and surrender of passport. (Bartosz)

Imprisonment was a trying time for friendships and acquaintances, which were no longer as advantageous for the other person as before the conviction. Two lawyers found themselves forsaken by their former colleagues, who did not wish to be associated with convicted criminals. In contrast, the politicians mostly maintained support from their parties and local communities, who often protested against their prosecution. Generally, the more powerful the subject, the stronger their bonds proved.

For many, the whole thing was embarrassing or even stinky. They weren't sure, they didn't know for sure if it was true, didn't want to get involved. I understand it and don't hold a grudge for it. (Michał)

Conversely, those who remained loyal often showed determination to improve the situation of their incarcerated friends. In two cases, the white-collar offenders were friends with lawyers, who were ready to go far above their paygrades and smuggled in the most needed items, brought news, or simply had a chat. Since such consultations did not count towards the limit of visits, these participants could eventually enjoy more contact with the world outside than their fellow inmates, who were mostly represented by underpaid public defenders. Powerful friends often advocated for the release of the white-collar offenders and the petitions and open letters signed by professional societies, public figures, and Catholic dignitaries were a common theme:

X stood up for me, you certainly know the name. The entire Club X vouched for me, where many notable figures were active. Professor X, then the Rector of the University of X, spoke out in my favor. That is, at least these people stood up for me and staked their good reputation on the fact that they did not believe the accusations. (Bartosz)

While Tomasz was being held in custody, his party organised protests in front of the court buildings. The gentlemen’s club to which Łukasz belonged gathered one afternoon at a site visible from his cell and began to sing and toast him. Both participants had no doubt that such incidents cemented the respect they enjoyed from both fellow inmates and prison staff. After the conviction of the white-collar offenders, their friends did not cease to support the imprisoned white-collar offenders materially and emotionally and it was often these informal connections that ensured them lucrative jobs after release.

## Discussion

Although ‘employing one’s specific personal capital to cope with adverse situations is unlikely to be specific to white-collar offenders’ (Hunter 2019), the volume and composition of that capital massively shaped the experience of custody of those in the sample. The imported conventional social resources mostly worked to the advantage of the white-collar offenders, although the conditions under which they were applied were moulded by structural factors such as prison type, calling for a caveat in favour of the pain of imprisonment theory (Sykes 1958). Notably, the largest differences between prison experiences within the sample consisted in the type of facility and not in variation of capital. Where the strict regime of remand forbade visitations and restricted access to prison jobs, social and cultural assets were not utilized to their full potential. However, once restrictions were lifted and opportunities emerged, the white-collar offenders were quick to avail of them; they actually used all visitations, regularly received rewards, and were assigned unchallenging but interesting jobs, often outside the facility. The following table contains a simplified roadmap of the four forms of capital in the three investigated fields. However, as capital constitutes both the means and the goals of interaction in a field (Bourdieau, 1983), the distinction between assets and ultimate benefits will inevitably be somewhat intuitive (Table 2).

**Table 2:** Summary of assets and derived benefits

		Interactions		
		Fellow Prisoners	Prison Staff	Family & Friends
Capital	Economic	[*primarily symbolic effect*] - material independence	not observed [*conceivable: bribery*]	- financial stability
	Social	- friendships with respected prisoners - groups of white-collar inmates	- fraternisation with educated staff	- strong familial bonds - connections to lawyers
	Cultural	- conversational skills - formal writing - guidance for juveniles	- understanding of rules - competencies useful for facility	- correspondence
	Symbolic	- image of criminal elite - admiration of riches - recognition of middle-class status	- status of “a normal person” - use of former titles	- belief in innocence - status retention
	Benefits	- physical safety - gratitude - cordiality - minor gifts & favours	- positive assessment - rewards & leaves - less strict control - desired jobs - practical advice - treatment as equals	- material help - legal advice - frequent visits - public advocacy - employment after release - contact with the outside

Own representation

### Economic capital

By the virtue of its strict regime, prison constitutes an enclave apparently unpermeated by the power of money. The access to financial means and their use were by and large denied to the inmates. Consequently, the economic hierarchy remained flat and did not play a central role in the structure of the field. Extra food products, cigarettes, and coffee were desired goods but did not distinguish their owners and convey power in the way described by Crewe (2009) with regard to heroin in an English facility. Accordingly, the transformation of economic capital into social capital was rarely successful, and sharing basic goods did not necessarily improve white-collar offenders’ position within the society of captives. The case in which Łukasz’s gift was taken for an insult by a cellmate illustrates the conversion risks characteristic of different sorts of capital, which cannot be directly compared to and exchanged for one another (Bourdieau, 1983). The limited relevance of economic capital to the prisoner hierarchy confirms the suitability of Bourdieusian theory, which places focus on other types of assets, for prison research. Similarly, Neuber (2011) argues physical violence among detained juveniles replaces other currencies by which their peers compete for status outside.

Nevertheless, the affluence of the participants exerted its effects through non-economic channels, namely as a symbolic resource. Many prisoners had access to coffee and cigarettes, but hardly any lived a wealthy life outside. The perceived thus trumped the tangible, and some white-collar offenders were revered as members of an alien financial elite, who were perceived to have also ‘made it’ outside prison. Fellow inmates were amazed by figures and asked awkward questions about the income and ways of ‘making money’. The findings highlight the difference between the primary and the symbolic effect of economic capital perceived through categories specific to the logic of the field (Bourdieu 1990). In the impoverished prison society, one was admired for being rich even though they had no access to their money, lived in the same cell, and ate the same food. Outside the carceral context, the participants’ economic capital was less constrained; it was used to hire top lawyers, who often secured an early release, covered the bail, and might have partly accounted for the stability of familial bonds.

### Social Capital

A series of studies have examined the role of social capital in prison adjustment and later desistance, although not all of them applied the Bourdieusian framework (Lafferty et al. 2016). As in other studies, good relations with friends and family were instrumental to a successful adjustment. Alongside emotional support, the relatives and close friends provided material assistance and covered the costs of legal counsel. Lawyer friends, in turn, illustrated Bourdieau (1983) assertion that the ‘quality’ of connections comes before their mere number. In possession of a rare institutionalised cultural asset (admission to the bar), such friends were able to do much more for their imprisoned friends than other prisoners could count on. It has been shown, however, that not every friend was still interested in investing in presently less profitable relations with a convicted offender (cf. Goldstraw-White 2012), which illustrates an idiosyncrasy of social capital, namely its dependence on other social actors. Unlike in the British studies (Button et al. 2018), spouses, who had already invested much in their relations with the subjects, largely stuck with their imprisoned partners. Friends were less likely to turn their backs on those participants who retained more individual assets. The reactions differed across fields – for the former colleagues of a prosecutor or a barrister, associating with an imprisoned convict would have apparently been synonymous with an all too heavy loss of symbolic capital.

Spending most of their time with other inmates and prison staff, the white-collar offenders built largely positive relations within the institution. The image of powerful criminals won them the recognition of the prisoner society, while their respectable appearance and compliance with the rules were appreciated by the staff. Therefore, the present results could explain why such offenders tend to have more friends in prison and experience fewer conflicts with cellmates, as was observed in previous studies (Stadler et al. 2013). The enclaves of the middle class inside prison, accumulating cultural and symbolic capital of their members, also increased the volume of each of their members’ social capital. With one exception, their self-identification as “not real criminals” did not lead the pragmatic white-collar offenders to distance themselves from other inmates and to lose informal support, as Hunter (2012) has suggested.

### Cultural Capital

Notwithstanding the special sensitivity argument, prisoners with a higher educational level are now believed to be better equipped to deal with the challenges of prison life (Crank & Payne 2015). The present results have shown that many middle-class cultural assets were, in fact, transferrable to the field of prison and worked in the favour of the white-collar offenders studied. Bourdieau (1983) praised this unique quality of incorporated cultural capital, which, unlike economic and social capital, exists within its holders, and cannot be taken from them. Accordingly, the white-collar offenders enjoyed the advantages of their knowledge, experience, and *savoir-faire* even under the conditions of isolation and severe material deprivation.

First, it allowed them to shape time in custody in a constructive way and avoid endemic boredom (cf. Payne 2003). Second, it rendered them considerate interlocutors, respectful cellmates, and good storytellers, who won the sympathy of fellow inmates. The kind of knowledge they possessed was valuable through its rarity, to which Bourdieu (1984) attributed a high power of distinction. Third, it enabled them to understand and follow the rules, sparing them potential conflicts with guards (cf. Benson & Cullen 1988; Crank & Payne 2015). Fourth, their formal writing skills and basic legal literacy proved useful for other prisoners less familiar with bureaucratic institutions (cf Hunter 2019; Button et al. 2020). Fifth, extensive experience in various fields could be employed for the facility, including for cultural programmes, rehabilitation, and legal matters. Sixth, in line with the assumption made by Shover and Hochstetler (2006: 138), cultural capital allowed the participants to secure favourable work assignments whilst incarcerated.

Turning to the idiosyncrasies of the prison field that have fueled the special sensitivity hypothesis, it must be noted that the prisoner society no more lived by the rules of ‘grypsera’ counterculture that rejected dominant culture in toto (Kaminski 2004) and nor were the participants entirely dismissive of the inmate culture (cf Benson & Cullen 1988). Adjustment required neither initiation rituals nor an extensive knowledge of argot and prison customs. Owing to their intellectual capacities, the white-collar offenders quickly picked up a functional knowledge of the prison field and used argot terms where appropriate.

### Symbolic Capital

The relevance of symbolic capital for white-collar crime reaches far beyond their adjustment to prison. Social status and reputation of white-collar offenders are thought to enable privileged offending and serve as a shield against effective prosecution (Sutherland 1983; Gottschalk 2020). This study shows how the effect of symbolic resources reaches into prison society and improves the standing of white-collar offenders within it.

In their interaction with guards, the status of ‘normal people’ constituted their main symbolic asset. By obeying prison rules and rejecting of deviant values, the white-collar offenders managed to negotiated and neutralise the criminal label (cf. Benson 1985; Benson & Cullen 1988) and escaped the infantilising regime of rehabilitation (cf. Mason 2007). Rather than becoming part of the mass of inmates, they won respect and narrowed the distance between them and the prison service, who gradually stepped out of their role and came to see the white-collar offenders as people who ‘got into prison by mistake’. The distinct forms in which the staff used to address the respondents, including their former professional titles, suggested a considerable volume of symbolic capital. As was shown, the trust of the guards was sometimes abused as in one respondent used his reputation to smuggle items into a cell and eventually went unpunished.

Even though the white-collar offenders studied belonged to a social class alien to the prisoner society, they were nevertheless convicted criminals. The degradation ceremonies aimed to undermine their public image actually had the reverse effect within prisoner hierarchies. As shown in the studies by Goldstraw-White (2012) and Huisman & Lesmeister (2018), the high publicity of cases, the seriousness of charges, and the large sums mentioned in arrest warrants served as a status enhancer, winning them the image of the criminal elite, and sparking the interest of fellow inmates. It must be noted, however, that conventional social values, such as education, were also a valid symbolic currency in most carceral settings, and the legitimate achievements of white-collar offenders were equally appreciated by their fellow inmates, even more so owing to their rarity.

## Conclusions

In terms of the importation hypothesis (Irwin 1970), the study at hand aimed to examine which assets exactly are imported by white-collar offenders into prison and how these assets exert their effect and retain relevance in the disparate field of prison. All four types of capital identified by Bourdieau (1983) shaped the social situation of the upper-world offenders studied and rendered them particularly resilient to prison hardship. The major conclusion is the immutable nature of privilege throughout the criminal career of a white-collar offender, reaching as far as the correctional system, which has been intuitively deemed a ‘hellish place’ for a businessperson or a politician.

The findings of this study can be taken/interpreted with the caveat that there are saturation limitations. The restrictive application of the offender-based definition came at the price of sample size, and, eventually, a total of 13 interviews were included. Additionally, the objective of presenting a cross-section of the upper-middle class was not fully achieved due to the lack of female white-collar offenders and convicted physicians in the sample. Finally, whilst the adopted theoretical framework‘s concepts aptly describe most aspects of the social situation of the subjects, they do not give adequate expression to the rich emotional and personal facets of the obtained accounts. These accounts, however, could still be fruitfully reanalysed from a more narrative framework.

Future studies could also focus on the experience of middle-class prisoners in general, who have been mentioned in major prison ethnographies (Goffman 1961; Crewe 2009) but have never received undivided scholarly attention. Coping with the label of a ‘conventional’ offence might be a challenge qualitatively dissimilar to the one white-collar offenders face. Research into imprisoned white-collar offenders would also benefit from the inclusion of Southern criminal justice systems, which have already imprisoned many corrupt politicians and public figures (Menaldo et al. 2021). Furthermore, the results point to a huge untapped potential of Bourdieu’s theory for inquiries into life in custody. In more general terms, the reflection on how societies should deal with those members of their elite who disregard the generally binding rules is much needed.

## Footnotes

1 but see: Huisman & Lesmeister 2018, Kotowska 2017.

2 This personal aspect is demonstrated in Pontell’s (2016) assertion that the patrician view ‘lowers the conceptual bar to the point where the original term becomes almost meaningless in that it produces a portrait of white-collar crime that includes a sizeable percentage of unemployed persons who have passed insufficient-funds checks at the local supermarket’.
